# Supplementary material for: Cyborg insect factory: automatic assembly for insect-computer hybrid robot via vision-guided robotic arm manipulation of custom bipolar electrodes
Source: Nat Commun. 2025 Jul 28;16:6073. doi: 10.1038/s41467-025-60779-1 (PMC12304280; doi:10.1038/s41467-025-60779-1)
Supplement: Supplementary file 1 — Supplementary Information [file 41467_2025_60779_MOESM1_ESM.pdf]

Supplementary Materials for

Title:

Cyborg Insect Factory: Automatic Assembly for Insect-computer Hybrid Robot via Vision-guided Robotic Arm Manipulation of Custom Bipolar Electrodes

Authors:

Qifeng Lin[1], Nghia Vuong[1], Kewei Song[1], Phuoc Thanh Tran-Ngoc[1], Greg Angelo Gonzales Nonato[1], Hirotaka Sato[1*]

Affiliations:
[1]School of Mechanical & Aerospace Engineering, Nanyang Technological University; 50 Nanyang Avenue, 639798, Singapore.

*Corresponding author. Email: hirosato@ntu.edu.sg

This file includes:

Fig. S1. Conventional implantation on the insect's antennae and cerci.

Fig. S2. Comparison between SCARA and 6DOF robot.

Fig. S3. Properties of bipolar electrode.

Fig. S4. Flowchart of the preparation for multiple insect-computer hybrid robots.

Fig. S5. Qualitative comparison of segmentation models on the cockroach dataset

Fig. S6. Qualitative comparison of TransUNet when trained on different loss functions.

Fig. S7. Qualitative comparison of TransUNet when trained on different data mixtures

Fig. S8. Stimulation sites with error of implantation.

Fig. S9. Assembly failure modes.

Fig. S10. Assembly for cockroach (6.0 – 7.0 cm) with different sizes of mounting structures.

Fig. S11. Four insect-computer hybrid robots covering obstructed terrain in 10 minutes with no stimulation applied.

Fig. S12. Covered area in one minute with different number of insects.

Table S1. Raw mechanical property parameters of the ABS-Like photopolymer

Table S2. Printing parameters of normal resin and active precursor used in this study

Table S3. Detailed parameters of the backpack

Table S4. Distance travelled and average speed of insects from control and experimental group in 10 minutes.

Table S5. Comparison of Direction Control.

Table S6. Locomotion Control Success Rate after assembly finished.

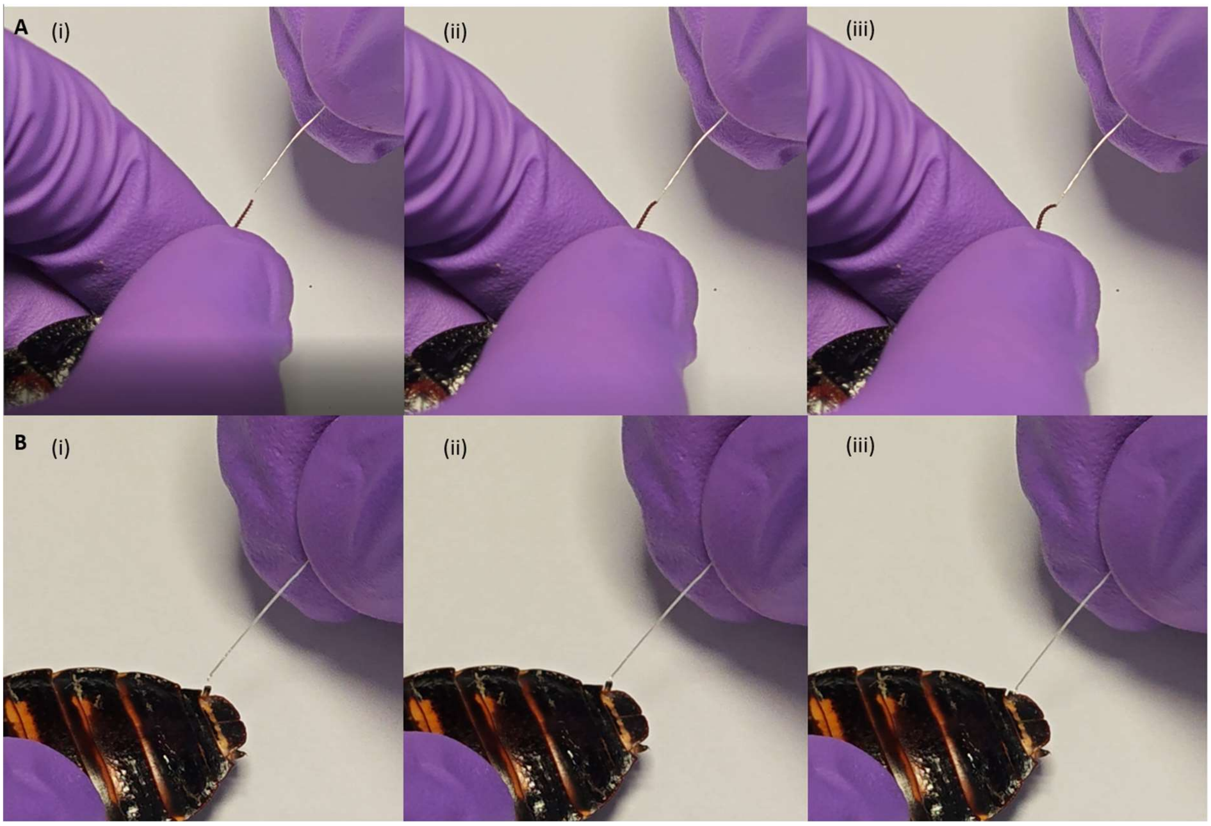

Fig. S1. Conventional implantation on the cockroach's antennae and cerci. (A) Silver wire implantation on the antennae. (i) Before implantation. (ii) Silver wire started inserting the cut antenna. (iii) Antennae changed shape during the silver wire inserting. **(B)** Silver wire implantation on the cerci. (i) Before implantation. (ii) Silver wire started inserting the cut cerci. (iii) Cerci shrunk during the silver wire inserting.

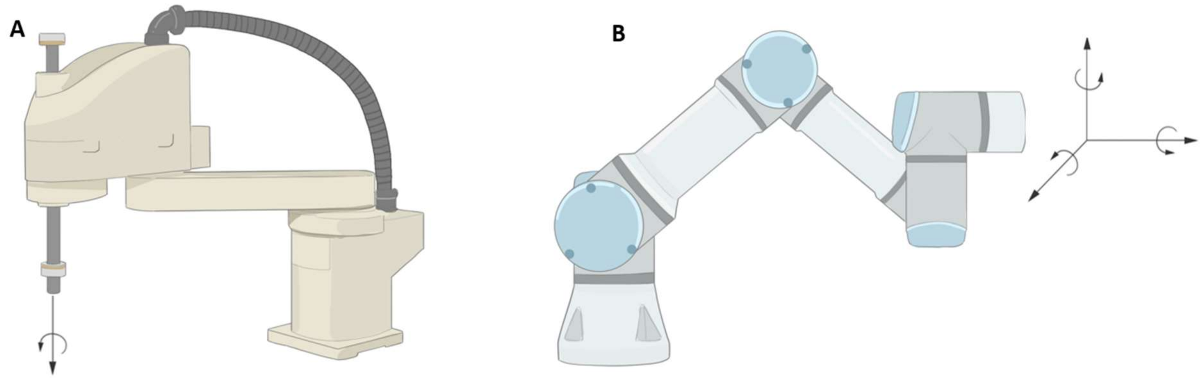

Fig. S2. Comparison between SCARA and 6DOF robot. (A) End effector of SCARA robot only has one freedom of rotation and cannot change the pitch angle flexibly. **(B)** End effector of 6DOF robot has three freedoms of rotation. During the study of the implantation pitch angle, 6DOF robot is more suitable.

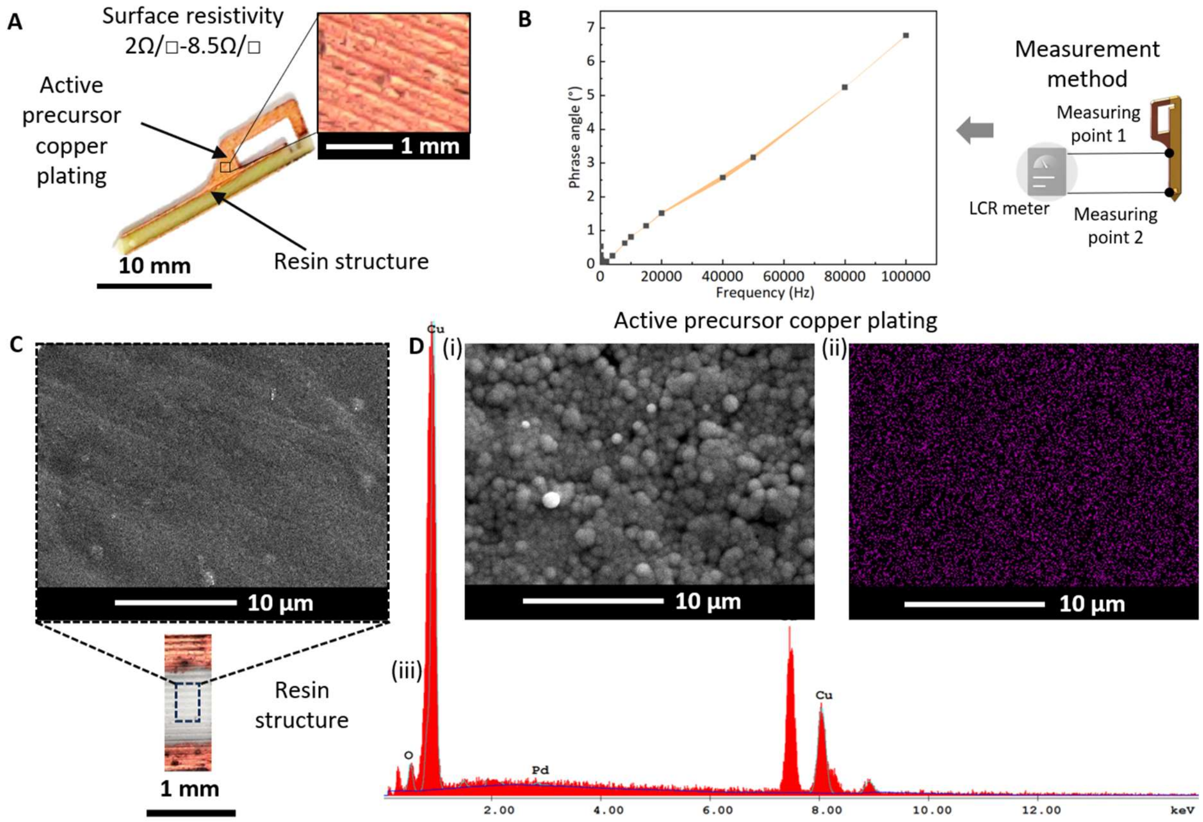

Fig. S3. (A) Schematic illustration of the active precursor copper plating process on the resin structure. The surface resistivity is measured to be between 2 Ω/□ and 8.5 Ω/□, indicating the successful formation of a conductive copper layer. The inset shows the surface morphology of the resin structure with a scale bar of 1 mm. **(B)** Phase angle vs. frequency. **(C)** SEM image of the resin structure prior to plating, displaying a relatively smooth surface with oriented microstructures, and the inset shows the sampling location within the resin structure (scale bar = 1 mm). **(D)** Characterization of the plated copper layer: (i) SEM image of the plated surface, showing a uniform granular structure with well-defined particle size distribution (scale bar = 10 µm); (ii) EDS elemental mapping of Cu, confirming the homogeneous distribution of copper over the plated surface; and (iii) EDS spectrum, identifying prominent Cu peaks, along with minor Pd and O peaks, indicating the successful catalytic activity of Pd and the formation of a stable copper layer.

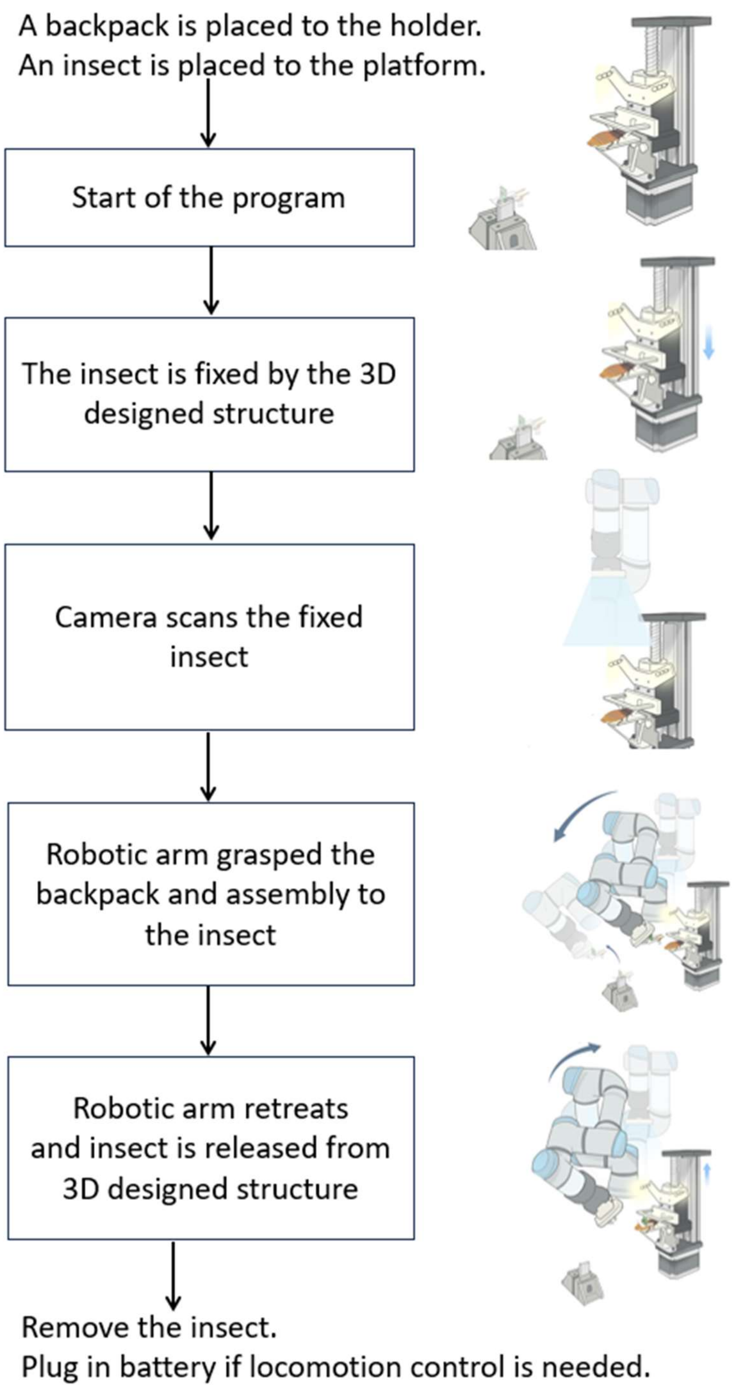

Fig. S4. Flowchart of the preparation for multiple insect-computer hybrid robots.

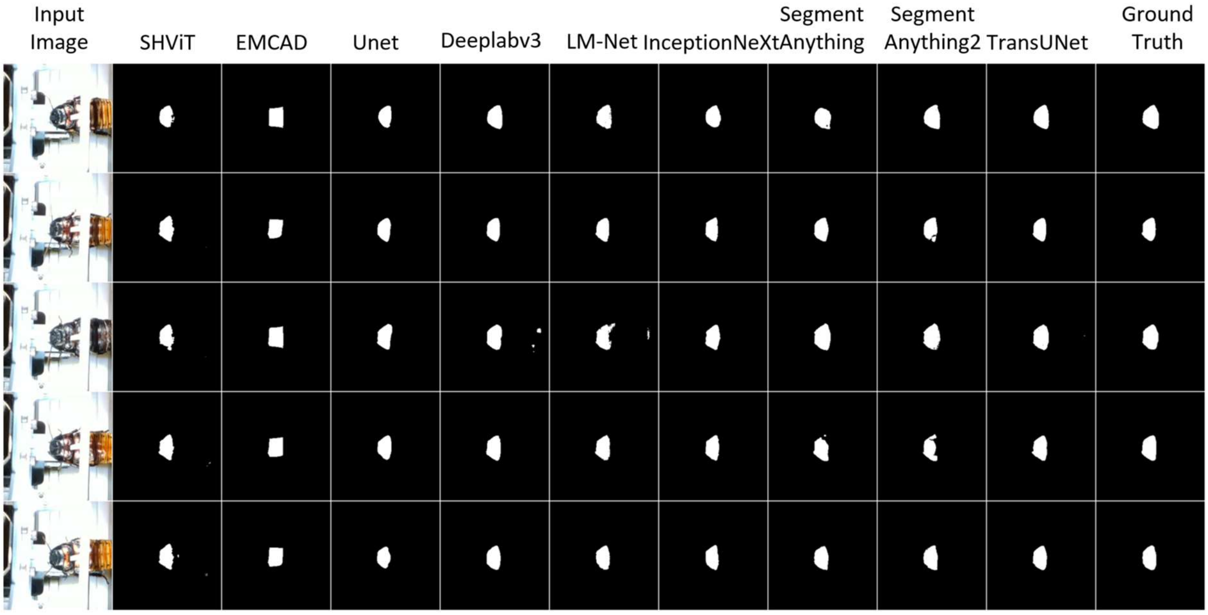

Fig. S5. Qualitative comparison of segmentation models on the cockroach dataset.

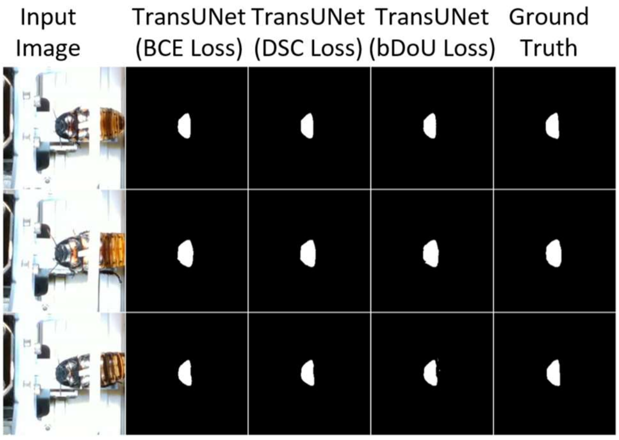

Fig. S6. Qualitative comparison of TransUNet when trained on different loss functions: BCE Loss, DSC Loss and bDoU Loss.

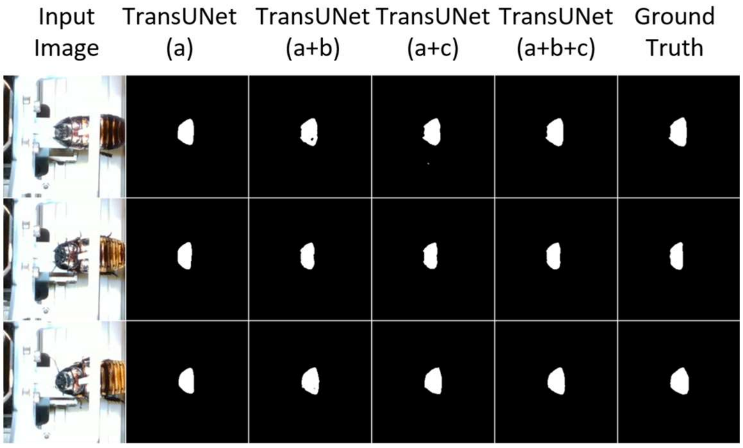

Fig. S7. Qualitative comparison of TransUNet when trained on different data mixtures.

(a) original unaugmented images, (b) asymmetrically scaled images, (c) rotated images

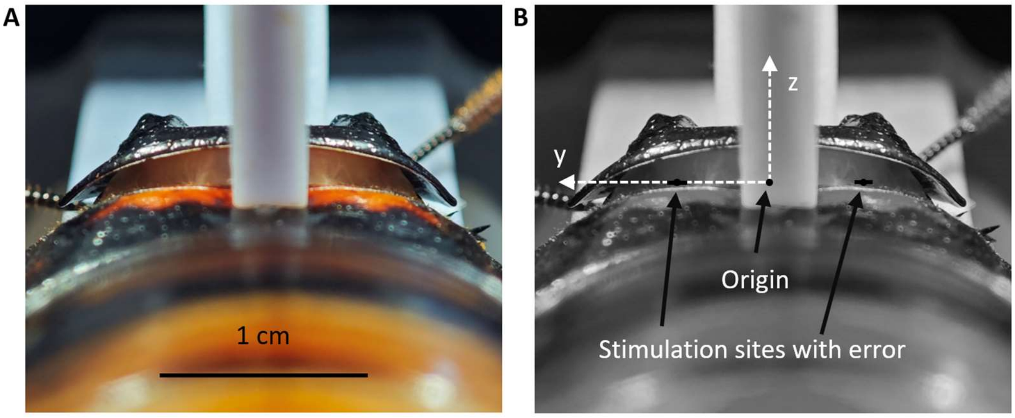

Fig. S8. Stimulation sites with error of implantation. As the origin detection has an error of 1.749 pixels, the real error for the implantation is 0.36 mm.

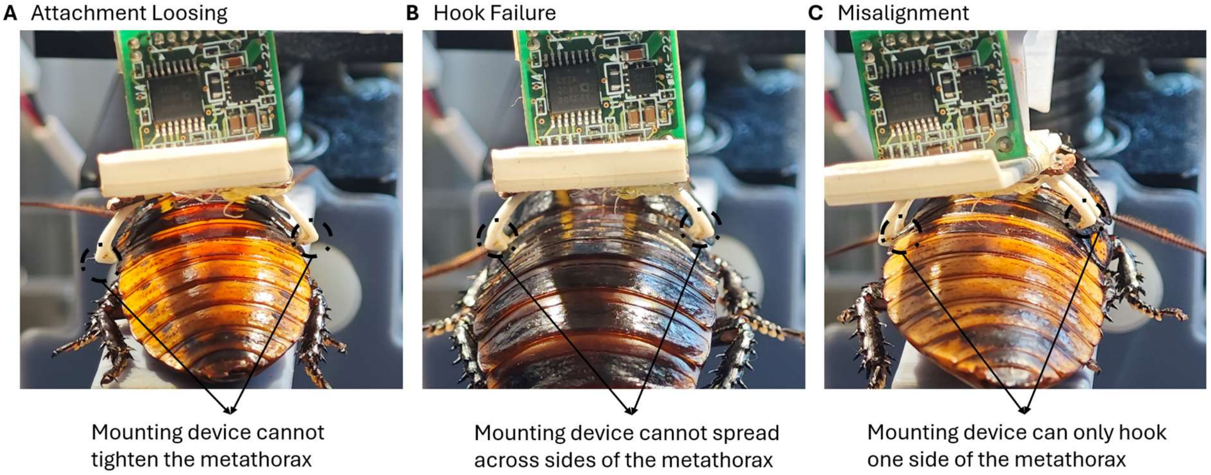

Fig. S9. Assembly failure modes. (A) Attachment loosing: mounting hooks did not tighten the insect’s metathorax. **(B)** Hook failure: no mounting hooks was attached to the insects. **(C)** Misalignment: mounting device was misaligned with the insect, resulting in only one side of the hooks being attached to it.

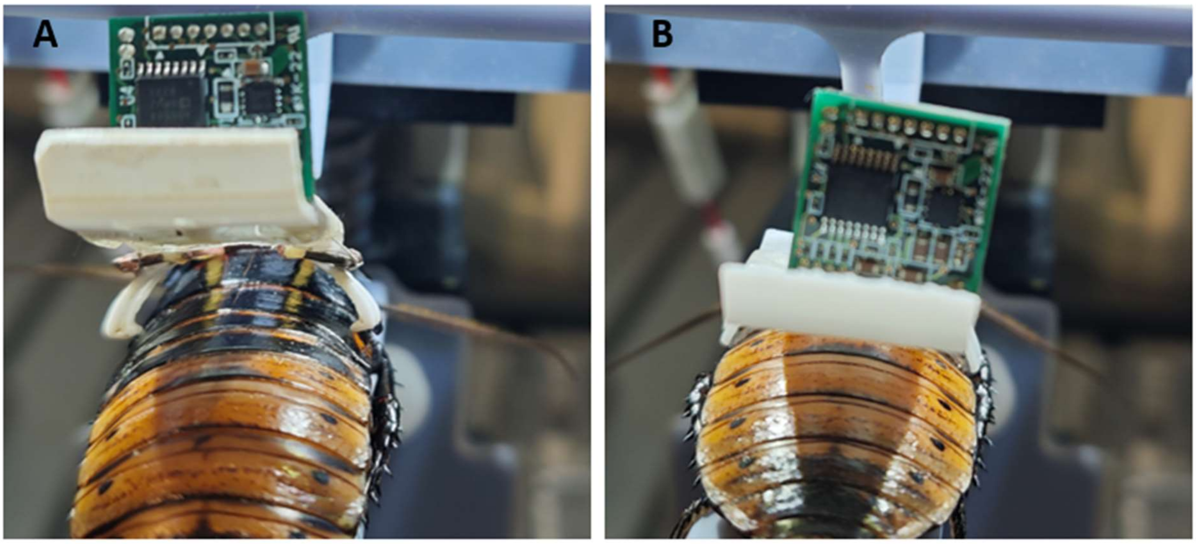

Fig. S10. Assembly for large size of cockroach (6.0 – 7.0 cm) with different sizes of mounting structures. The enlarged hook structure (1 mm enlarged for each hook branch) improved success rate of automatic assembly from 46.7% (6.0 – 6.5 cm) and 13.0% (6.5 – 7.0 cm) to 80.0% (6.0 – 7.0 cm). **(A)** Hook failure with normal size of mounting structure. **(B)** Success with enlarged size of mounting structure.

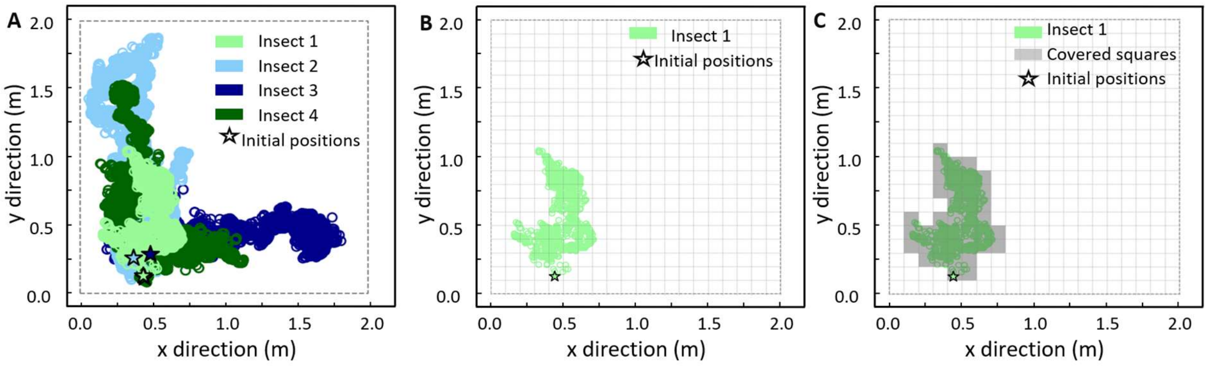

Fig. S11. **(A)** Four insect-computer hybrid robots covering obstructed terrain in 10 minutes with no stimulation applied. **(B)** Insect 1 was used for calculation of its covered area. The target terrain was divided into 20 * 20 squares. **(C)** The squares were labelled as "covered" when the insects passed. 40 squares were covered in total by Insect 1. Hence, 4000 cm^2 was covered by Insect 1.

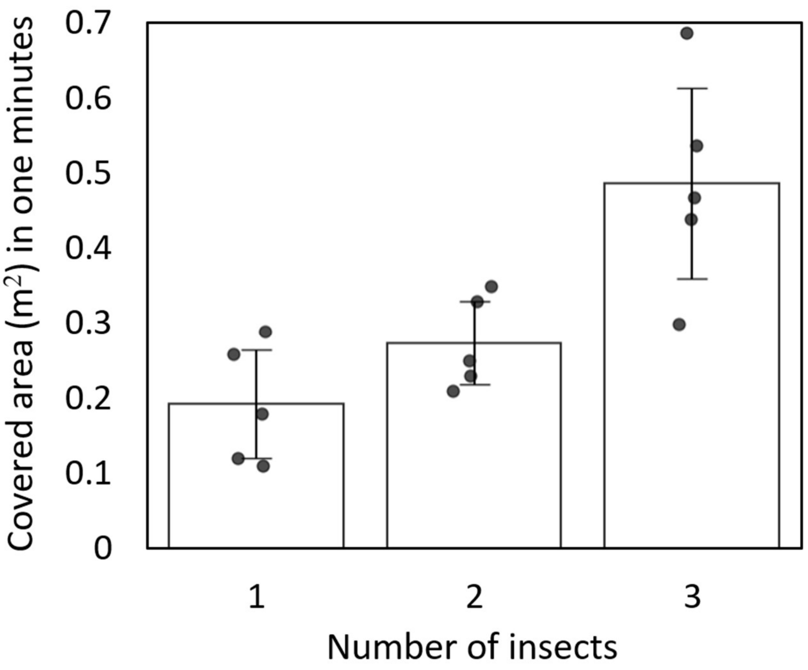

Fig. S12. Covered area in one minute with different numbers of insects. With the number of insects increasing from one to three, the average covered area increased from 0.19 m^2 to 0.49 m^2. For each number of insects, 5 trials of the experiment were conducted. The error bars denote the standard deviation.

Table S1. Raw mechanical property parameters of ABS-like photopolymer used in this study

Items	Standard	Value
Model	/	603A
Attributes	/	Tough resin
Appearance	/	multiple colour
Viscosity	（cps@25℃）	260 - 350
Critical exposure energy	(mJ/cm2)	8.1 - 9.0
Tensile Strength (MPa)	ASTM D638	46
Tensile modulus (MPa)	ASTM D638	2200
Bending strength (MPa)	ASTM D790	64
Flexural modulus (MPa)	ASTM D790	1900
Elongation (%)	ASTM D638	15 - 25
Notched impact strength (J/m)	ASTM D256	40
Heat distortion temperature (℃)	ASTM D 648 @66PSI	63
Shore hardness (D)	/	75

Table S2. Printing parameters of normal resin and active precursor used in this study

Resin type	Single layer exposure time	Slice thickness
Normal resin	6 - 11 s	0.05 mm or 0.1 mm
Active precursor	8 - 12 s	0.05 mm or 0.1 mm

Table S3. Detailed parameters of the backpack

Backpack elements	Length (mm)	Width (mm)	Height (mm)	Volume (mm^3)	Weight (g)
Mounting structure	32.0	21.4	14.6	575.5	0.8
Bipolar electrode	20.7	5.0	1.6	31.3	0.05
Microcontroller	15.0	15.0	17.0	787.5	1.4

Table S4. Distance travelled and average speed of insects from control and experimental group in 10 minutes.

Group	Insect No	Distance travelled (m)	Average speed (cm/s)
Control group	1	62.9	10.5
	2	65.3	10.9
	3	64.4	10.7
	4	56.3	9.4
Experimental group	1	83.8	13.9
	2	95.8	15.9
	3	108.4	18.1
	4	102.2	17.0

Table S5. Comparison of Direction Control. The code for motion capture is available at https://github.com/Qifeng-Lin/MotionCapture.

Stimulation method	Average turning angle	Stimulation success rate	Survival rate
Abdomen-based	20.6°	76%	100%
Antenna-based	66.6°	98%	100%
Pronotum-based	75.2°	100%	100%

Table S6. Locomotion Control Success Rate after assembly finished.

Assembly Method	1 hour	4 hours	8 hours
Automatically assembled	100%	91.7%	91.7%
Manually assembled	100%	83.3%	100 %
