# Supplementary material for: Cyborg insect factory: automatic assembly for insect-computer hybrid robot via vision-guided robotic arm manipulation of custom bipolar electrodes
Source: Nat Commun. 2025 Jul 28;16:6073. doi: 10.1038/s41467-025-60779-1 (PMC12304280; doi:10.1038/s41467-025-60779-1)
Supplement: Supplementary file 2 — Description of Additional Supplementary Files [file 41467_2025_60779_MOESM2_ESM.pdf]

Description of Additional Supplementary Files

File name: Supplementary Movie 1
Description: Preparation for the bipolar electrodes. Multi-material DLP3D printing and electroless plating were used to manufacture bipolar electrodes. Copper was deposited on the sides of the designed electrodes to transfer the electrical stimulation to the insects.

File name: Supplementary Movie 2
Description: Front legs movement of the insect-computer hybrid robot during stimulation. When the right turning stimulation happens, the left front leg was stimulated to contract and vice versa. When the deceleration stimulation happens, both front legs were stimulated to contract. The contract of front legs shows the insect was under stimulation, indicating that the insect's turning motion was directly due to the stimulation of the associated front leg.

File name: Supplementary Movie 3
Description: Locomotion control of insect-computer hybrid robot. Insect-computer hybrid robot was stimulated to steer and decelerate.

File name: Supplementary Movie 4
Description: Automatic assembly of the insect-computer hybrid robot. Insect was firstly fixed by a 3D structure driven by a slide motor. After fixation, its intersegmental membrane between the pronotum and mesothorax was exposed. Gripper of the robotic arm was then activated, and the RealSense Camera scanned the insect to find the specific position of the reference point, pR, on the edge of the pronotum. Afterwards, robotic arm went down to carry the backpack and implanted the bipolar electrodes to the exposed intersegmental membrane. Branches of the backpack were than pressed to fix the backpack to the insect. Finally, robotic arm and slider motor went back to the home position for next assembly. One insect-computer hybrid robot was assembled successfully.

File name: Supplementary Movie 5
Description: S-shape line following of the insect-computer hybrid robot.
